# Role of Long-Chain Cyanoacrylate as an Adjunct Interposition Layer in Repair of Hypospadias and Urethrocutaneous Fistula in Children: A Novel Technique

**DOI:** 10.7759/cureus.57693

**Published:** 2024-04-05

**Authors:** Ina Bahl, Sanat Kumar Khanna, Saurabh Maheshwari

**Affiliations:** 1 General Surgery, Command Hospital, Chandimandir, IND; 2 Pathology and Laboratory Medicine, Command Hospital, Chandimandir, IND

**Keywords:** hypospadias surgery, pediatric reconstructive urology, adhesive agent, urethral-cutaneous fistula, hypospadias repair

## Abstract

Purpose

Hypospadias is an anomaly wherein the urethral opening is ectopically located on the ventral aspect of the penis. The most common complications after hypospadias repair are urethrocutaneous fistula (UCF) and meatal stenosis. Long Chain Cyanoacrylate (LCCA) tissue adhesive promises safety, feasibility, and durability due to its tensile strength and bacteriostatic and hemostatic properties. We conducted this study to ascertain whether LCCA tissue adhesive can prove a more effective adjunct to traditional suturing techniques.

Methods

Patients were divided into two groups. Group A underwent surgery with conventional reconstruction of the neourethral tube along with the buttressing layer using Buck's fascia or Tunica Vaginalis. In addition to the traditional procedure of Group A, Group B patients were administered a layer of LCCA tissue adhesive as an adjunct between the neourethral suture line and the buttressing layer. Patients were followed up for six months and were evaluated for complications like UCF, meatal stenosis, hematoma, skin infection, glans dehiscence, and flap necrosis.

Results

Thirty-eight children in the age group 1-6 years were studied, of which 20 were in Group A and 18 in Group B. Among patients of Group A seven (35%) developed complications. In contrast, only four (22.2%) patients developed complications in Group B. The statistical significance in the complication rates between the two groups could not be achieved due to the modest sample size. However, the numerical and proportional reduction in the number of complications was noted.

Conclusion

LCCA adhesive as an adjunct numerically reduces the number of complications compared to traditional suturing alone in patients undergoing surgery for hypospadias and UCF.

## Introduction

Hypospadias is the second most common congenital anomaly of the genital system in boys, following cryptorchidism [1]. It is an anomaly of penile development, wherein the urethral opening is ectopically located on the ventral aspect of the penis, associated with chordee and deficiency of prepuce [2]. Correction of hypospadias is one of the most common procedures in pediatric surgery and pediatric urology [1]. As is the case with pediatric anomalies, hypospadias poses a surgical challenge. It imposes a socioeconomic burden on the family, causing paramount anxiety regarding long-term cosmetic and reproductive outcomes among parents and adolescents alike. Multiple surgical options and algorithms are available to guide management based on the classification of hypospadias [3].

Surgical procedures can either be stratified based on the number of surgeries as one-staged and two-staged or the type of surgery into urethral plate tubularization, augmentation, and replacement [4]. Complication rates following hypospadias surgery are high. Post-operative complications can usually be identified early, in the first few months after surgery in most cases. Still, long-term follow-up is mandatory because delayed presentation with a urethral fistula or meatal stenosis is well known. The common complications after hypospadias repair include urethrocutaneous fistula (UCF), meatal stenosis, narrow tubularized urethra, urethral strictures, and residual chordee [5]. Amongst these, UCF is the most common complication of hypospadias repair, with a reported incidence of 4-25% depending on the severity of hypospadias, surgical technique, and experience of the operating surgeon [6].

The outcome is most satisfactory for all reconstructive procedures after the first surgical procedure [4]. Hence, the need of the hour is to devise an ingenious method of correction that ensures both cosmesis and definitive repair. Cyanoacrylate tissue adhesive is a new and promising advent due to its tensile strength and bacteriostatic and hemostatic properties. An ideal closure method in children should be painless and rapid, easy to perform, safe, with fewer complications, and cause minimal scarring [7, 8]. Closure with conventional suturing may not accomplish these objectives, which may be achieved using tissue adhesives. 

Studies on the use of cyanoacrylate tissue adhesive in pediatric urology are limited. It has been used to repair a surgical incision, wound closure in circumcision, and repair of UCF post hypospadias correction. However, few studies have tested the utility of cyanoacrylate derivatives as an alternative to traditional surgical treatment of hypospadias in children, especially in the Indian population. With this background, we aimed to study the safety and efficacy of Long Chain Cyanoacrylate (LCCA) as an adjunct to suturing technique for hypospadias and UCF repair in children by analyzing the post-operative complications and adverse effects within six months of surgery.

This article was previously posted to the Research Square preprint server on 05 May 2022. The preprint version is not pending full publication elsewhere.

## Materials and methods

Study design

A prospective study was conducted on children with hypospadias and urethrocutaneous fistula in a single tertiary care center. Patients were divided into two groups. Group A comprised boys who underwent surgery with the conventional suturing technique, and Group B included patients who underwent repair using LCCA as an adjunct to the traditional suturing technique. Effectively, boys in both Group A and Group B underwent suturing techniques for the reconstruction of a neourethral tube as well as a second layer of buttressing using Bucks fascia or tunica vaginalis. In addition, however, boys in Group B underwent an additional step in the form of layering of the neourethra with LCCA between the tube and the buttressing layer.

The duration of the study was 18 months (June 2020 to December 2021). The inclusion criteria were all pediatric patients with hypospadias of any severity. All boys were age-matched for the grade of severity of hypospadias. Patients with post-operative UCF were also included. Children with chronic diseases of any type that might interfere with the normal healing process were excluded. Further, those children whose parents did not consent to the study were excluded.

Method of selection

The patients were evaluated in detail, and the parents were counseled about the procedure pre-operatively regarding the risks and benefits of the surgical procedure. The diagnosis of hypospadias was established in all patients based on history and physical examination. Before surgery, written informed consent for surgery was taken from the patients' parents. The patients were assigned to Group A and Group B by simple randomization.

Technique

All surgeries were carried out under general anesthesia with due precautions regarding intra-operative antibiotic administration and post-operative wound and urethral stent care. Children were catheterized using 6 to 8 French (Fr) feeding tubes (as per age) over which the urethral tube was constructed, and this catheter was removed on the 10th postoperative day. Surgery for hypospadias was performed in both groups, which included a traditional suturing technique followed by a second buttressing layer using either Buck's fascia or the ipsilateral tunica vaginalis.

Children with severe chordee and proximal hypospadias underwent staged repair. Children with distal and mid-penile hypospadias underwent Snodgrass tubularized incised plate (TIP) urethroplasty. In the case of UCF, the fistulous area was prepared with gentle scarification of the edges and the edges of the fistula were approximated with a 6 − 0 polydioxanone suture.

**Figure 1:**
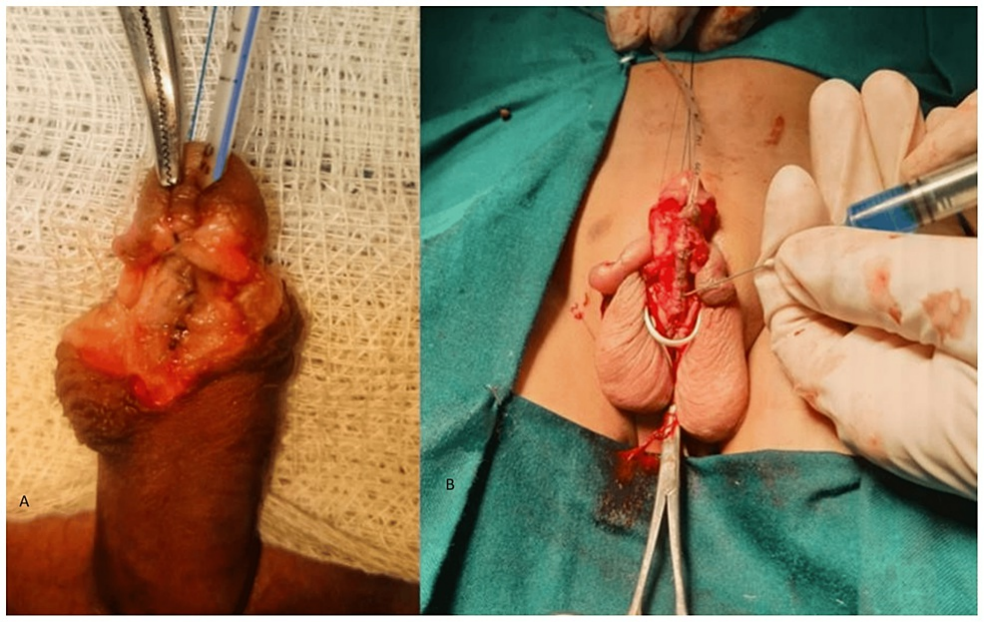
A: Intra-operative photographs of neourethral tube from the cases in Group A; B: Intra-operative photographs from Group B where LCCA is used as an adjunct LCCA: long chain cyano-acrylate

**Figure 2:**
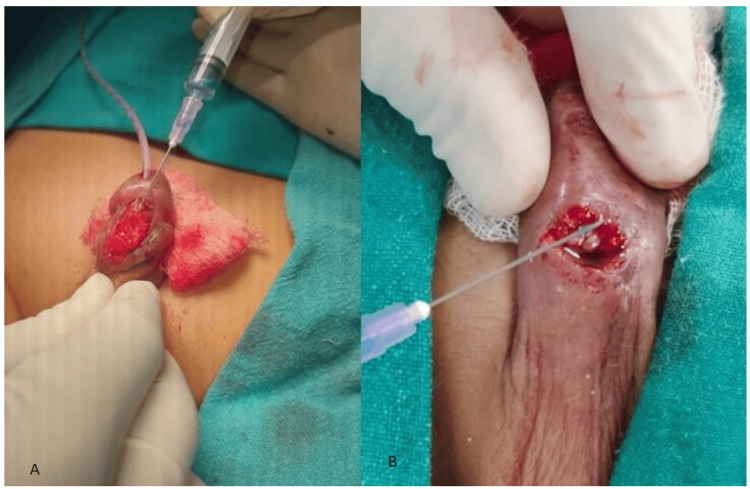
Intraoperative pictures of adhesive application during urethroplasty (A) and UCF repair (B) UCF: urethrocutaneous fistula

In Group B, following the traditional surgical suturing technique, the patients underwent an additional layer of application of varying quantities (1-2 ml) of LCCA between the reconstructed tube and the buttressing layer. On drying of adhesive, after 30 seconds, the dried flakes of extra adhesive on the edges were carefully removed with toothed forceps, followed by the buttressing layer of Buck's fascia or tunica vaginalis. Finally, glansplasty and the closure of the wound were done. In the UCF patients included in Group B, LCCA adhesive (0.2-0.5 ml) was applied to the edges of the UCF after traditional suturing to approximate the edges of the UCF. The dressing was done with antibiotic ointment and paraffin gauze in both Groups. The intra-operative clinical photographs of some of the cases encountered in the study are illustrated in Figures 1,2.

Assessment of objective outcome

On day three after surgery, the dressings were removed, and the wound was inspected. After 10 days of surgery, the children were discharged if the post-op stay was uneventful and were followed up for six months. The first visit was after seven days of discharge and subsequent monthly follow-ups. Patients were evaluated for complications like hematoma, skin infection, glans or urethral tube dehiscence, skin necrosis, meatal stenosis, and urethrocutaneous fistula on each visit.

Statistical evaluation

The descriptive phase of the analysis includes the presentation of data as raw proportions and means and standard deviations. Fisher's exact test was used to compare qualitative variables. The distribution of the quantitative variables was analyzed with the Kolmogorov-Smirnov test to determine whether they showed a normal distribution as required for the use of the Student's t-test. Statistical Package for the Social Sciences (SPSS) version 27.0 (IBM Corp., Armonk, USA) was used for data processing and analysis. For all variables, a p-value < 0.05 was considered statistically significant.

Ethics approval and consent to participate

The present study was approved by the ethical board of the hospital in which the study was performed. The patients reported in this article had signed a written informed consent form. This study was conducted in a medical, educational center. All patients were informed that they may be subjects of scientific experiments with the preservation of confidentiality and were informed of the ethical codes of conduct. This study complied with the latest version of the Declaration of Helsinki.

## Results

A total of 38 children were included in the study. This included 20 patients in Group A and 18 patients in Group B. Of the 20 patients in Group A, 14 had hypospadias, while the remaining six presented as UCF. Similarly, in Group B, 14 of the 18 patients presented with hypospadias, while the remaining four had UCF. The mean age of all 38 patients was 3.2 years (Range 1-6 years). The mean age was 3.1±0.3 years in Group A and 3.5±0.3 years in Group B. Children in both groups were age-matched for the type of hypospadias (according to severity). There was no statistical difference in age between the two groups (p-value = 0.35). Distal hypospadias was the most common type encountered in the study, seen in 11 (55%) and 12 (66.6%) cases in Group A and Group B, respectively. Mid-penile and proximal types were the least common hypospadias (Table 1). Mid-penile hypospadias was seen in one patient in Group A and Group B, while the proximal type (penoscrotal) was seen among two patients in Group A and a single patient in Group B. All three patients with the penoscrotal type of hypospadias had associated severe chordee. There was no significant difference in the types of hypospadias included in the two Groups (p-value =0.99). Similarly, there was no significant difference in the number of postoperative UCF patients included in Groups A and B.

**Table 1:** Distribution of types of patients in each group UCF: urethrocutaneous fistula

Type of patients	Group A (n=20) n (%)	Group B (n=18) n (%)	p value
Hypospadias	14 (70%)	14 (77.8%)	0.99 (Fisher’s exact test)
Distal:	11 (55%)	12 (66.6%)	
Glanular	-	-	
Coronal	6 (30.0%)	7 (38.8%)	
Subcoronal	3 (15.0%)	3 (16.7%)	
Distal penile	2 (10.0%)	2 (11.1%)	
Mid penile:	1 (5.0%)	1 (5.6%)	
Proximal:	2 (10.0%)	1 (5.6%)	
Scrotal	-	-	
Penoscrotal	2 (10.0%)	1 (5.6%)	
Perineal	-	-	
UCF	6 (30%)	4 (22.2%)	

In Group A, seven (35%) patients developed post-procedure complications. In comparison, only four (22.2%) patients had complications in Group B. There was a numerical reduction in the complication rate while using the LCCA adhesive as an adjunct, imparting a scientific impetus to the study. However, the modest sample size could not achieve statistical significance (p=0.272). Various complications observed in our study are depicted in Table 2. Three cases developed more than one complication (two patients in Group A and a solitary case in Group B). The most common complication was the development of UCF, which was seen in five (25%) patients in Group A and three (16.6%) patients in Group B. The least common complication encountered was dermal necrosis which was seen in one of the patients in Group A. In neither of the Groups, patients developed glans dehiscence or hematoma. Two patients in Group A and one in Group B developed meatal stenosis. One patient in each group had a wound infection. The number of patients presenting with complications during follow-up at various time frames is illustrated in Table 3. The minimum time for the development of UCF was two weeks. The maximum number of complications occurred within a month after surgical repair (six in Group A and two in Group B). The patient who developed dermal necrosis was managed conservatively. Two patients who had wound infections were managed with serial dressings, of whom one required administration of intravenous antibiotics according to culture sensitivity. Of the eight patients who developed UCF, five (three in Group A and two in Group B) were successfully managed with the help of calibration. The remaining three patients of UCF (two of Group A and one of Group B) had to be re-operated within 3 to 6 months of the development of UCF. None of the patients developed allergic or adverse systemic or hemodynamic effects due to the use of LCCA.

**Table 2:** Distribution of complications in each group

Complications	Group A (n=20) n (%)	Group B (n=18) n (%)	p value
Glans Dehiscence	0 (0%)	0 (0%)	0.272 (Chi-square test)
Urethrocutaneous fistula	5 (25%)	3 (16.6%)	
Meatal stenosis	2 (10%)	1 (5.5%)	
Dermal necrosis	1 (5%)	0 (0%)	
Hematoma	0 (0%)	0 (0%)	
Wound infection	1 (5%)	1 (5.5%)	
Total	9 (45%)	5 (27.7%)	

**Table 3:** Time elapsed before appearance of complications (in weeks)

Postsurgical duration (weeks)	Group A (n=9) n (%)	Group B (n=5) n (%)	p value
0-4	6 (66.6%)	2 (40%)	0.99 (Fisher’ exact test)
5-8	0 (0%)	1 (20%)	
9-12	2 (22.2%)	0 (0%)	
13-16	1 (11.1%)	0 (0%)	
17-20	0 (0%)	1 (20%)	
19-24	0 (0%)	0 (0%)	

## Discussion

Hypospadias is one of the most common congenital anomalies encountered in pediatric surgical practice [1]. It is a condition wherein the urethral opening is ectopically located on the ventral aspect of the penis. It is frequently associated with ventral penile curvature. The most common type of hypospadias in our study was coronal, which was seen in 13 (34.2%) cases, which is consistent with the findings of a study by Gamal Al-Saied et al [9]. Surgical intervention for hypospadias can be performed at any age; however, most authors recommend operative intervention at 6-18 months [10]. The mean age of surgery in our study was 3.1±0.3 years and 3.5±0.3 years in Group A and Group B, respectively. Although it is one of the most common surgeries performed by pediatric urologists, the high rates of complications, especially UCF, make it a challenge. In fact, multiple surgeries are required in more than 15% of children with hypospadias [11]. The most common complication which requires reoperation is UCF [12].

Reoperations and complications lead to a financial burden on the family, surgery and anesthesia-related complications, and long-term psychosexual issues. Alternate measures, including temporary re-catheterization and the use of tissue sealants, have been explored to reduce the complication rate of hypospadias surgery [7]. Butyl and octyl cyanoacrylate have been used extensively in the primary closure of pediatric surgical wounds, including sutureless circumcision and lacerations [7]. Tsur et al were the first to describe butyl cyanoacrylate in combination with sutures for hypospadias repair in the 1970s, which was later reinforced by Lapointe et al in their experience with butyl cyanoacrylates for fistula closure after hypospadias repair [13,14]. The most important advantages of using cyanoacrylate adhesive are its ease of application, tensile strength, and hemostasis conducive to preventing complications. In the present study, all patients underwent urethroplasty by traditional suturing technique. Group B patients also underwent adjunctive adhesive application. The glue used in the present study was n-butyl cyanoacrylate.

The most common complications following hypospadias repair are the occurrence of UCF, edema, and penile torsion [11]. In our study, the frequency of development of UCF was five (25%) in Group A and three (16.6%) in Group B. This is in concurrence with a study by Saroj C Gopal et al, where a total of 120 patients with proximal penile hypospadias were studied, in which it was found that 10% of patients in whom fibrin glue was used developed UCF. In contrast, 32% of the patients developed UCF in whom no glue was used [15]. Four comparative studies were included in the meta-analysis performed by A Singh et al [16]. They observed that the use of adhesives did not statistically reduce overall complication rates, which is consonant with the index study (RR 0.63, p-value = 0.13). However, they found a significant reduction in urethrocutaneous fistula (RR 0.37, p = 0.003), complications involving the neo-urethra (RR 0.15, p =0.004), and wound-related complications (RR 0.57, p = 0.008). In another study by G Ambriz-González et al [17], UCF was significantly lesser with adhesive than in the suturing-only group (10% vs. 41%). We also observed that UCF as a complication occurred in fewer patients where the adhesive was used as an adjunct.

In our study, the incidence of meatal stenosis was two (10%) in Group A and one (5.5%) in Group B, which was relatively higher vis-a-vis the study by Yuhao Wu et al where the incidence was 2.1% [18]. Dermal necrosis was noted in one patient in Group A in our study. A similar study by Hosseini et al revealed necrosis in 10% of patients who underwent urethroplasty with adjunctive adhesive [8]. None of the patients in our study had penile torsion following surgery, while the incidence of penile torsion following urethroplasty ranges from 1.7% and 27%, according to studies conducted worldwide [19]. Lapointe et al, in their study, primarily used adhesive to successfully treat six out of 13 patients with postsurgical UCF [14]. In our study, we also managed four patients with UCF by using adhesive as an adjunct (Group B), of whom two cases recovered without any recurrence.

The present study's findings, in general, corroborate with studies conducted worldwide. However, as discussed, certain findings were at variance with the results and conclusions of a few other studies. These differences could result from a smaller sample size, differences in the surgical techniques followed amongst different institutes, and a relatively shorter follow-up period of our study. In our study, we did not encounter any form of allergic or adverse hemodynamic effects from the glue. LCCA tissue adhesive proved to be a safe and effective solution to this overwhelming number of complications.

## Conclusions

LCCA has shown promising results in hypospadias repair due to its ease of application and greater tensile strength. It seems to confer a more significant advantage in the repair of hypospadias and UCF repair because of its biophysical properties, improved safety profile, and ease of intra-operative application with subsequently reduced rates of complications compared to surgical repair alone. By reducing complications such as UCF and meatal stenosis, the patients and parents are saved from the emotional trauma and the costs associated with redo procedures. The authors recommend that the results of the present study be adopted for further analysis using large-scale randomized control trials to allow a more accurate and meaningful comparison of results.
